# Modelling Childhood Growth Using Fractional Polynomials and Linear Splines

**DOI:** 10.1159/000362695

**Published:** 2014-11-18

**Authors:** Kate Tilling, Corrie Macdonald-Wallis, Debbie A. Lawlor, Rachael A. Hughes, Laura D. Howe

**Affiliations:** School of Social and Community Medicine and MRC Integrative Epidemiology Unit, University of Bristol, Bristol, UK

**Keywords:** ALSPAC, Growth, Linear spline models, Longitudinal study, Multilevel models, Repeated measures

## Abstract

**Background:**

There is increasing emphasis in medical research on modelling growth across the life course and identifying factors associated with growth. Here, we demonstrate multilevel models for childhood growth either as a smooth function (using fractional polynomials) or a set of connected linear phases (using linear splines).

**Methods:**

We related parental social class to height from birth to 10 years of age in 5,588 girls from the Avon Longitudinal Study of Parents and Children (ALSPAC). Multilevel fractional polynomial modelling identified the best-fitting model as being of degree 2 with powers of the square root of age, and the square root of age multiplied by the log of age. The multilevel linear spline model identified knot points at 3, 12 and 36 months of age.

**Results:**

Both the fractional polynomial and linear spline models show an initially fast rate of growth, which slowed over time. Both models also showed that there was a disparity in length between manual and non-manual social class infants at birth, which decreased in magnitude until approximately 1 year of age and then increased.

**Conclusions:**

Multilevel fractional polynomials give a more realistic smooth function, and linear spline models are easily interpretable. Each can be used to summarise individual growth trajectories and their relationships with individual-level exposures.

## Introduction

Childhood growth is increasingly seen both as an important outcome [1] and an exposure [2] or mediator for later-life outcomes. In order to investigate the factors which influence growth, or the outcomes with which it is associated [3], childhood growth needs to be accurately modelled. Analysis of a repeated outcome, such as weight or height, needs to take the correlation between repeated observations on the same person into account [4]: methods to do this [e.g. multilevel models (MLMs)] are now widely available in standard statistical software packages [5]. Measurement error may vary over time (e.g. absolute measurement error in weight will be larger in later childhood than at birth) and there will often be dropout due to non-response, illness or emigration, for example.

MLMs are often used to describe trajectories, as they analyse repeated measures (level 1) clustered within individuals (level 2). One approach is to model height as a simple function of age (which could be linear or could include polynomials). The aim of this paper is to describe two methods for choosing the ‘best-fitting’ trajectory within the multilevel modelling framework using an illustrative example. The first method is that of fractional polynomials, which can be used to generate a smooth function of growth with age. The second method is that of linear splines, which generate a set of connected phases, each with a different linear growth rate. We apply these models to data we have previously analysed using these methods on height growth in girls during childhood, compare the models and show how they can be used to examine the association between an exposure (in our example, parental social class) and growth. This example is used to illustrate the methods, and is not intended to be interpreted as an estimate of the causal effect of parental social class on growth – we have not considered confounding or any potential sources of bias. We have used height in girls as an example here, but these methods could be applied to any scenario where a continuous outcome is measured repeatedly in a group of individuals.

## Summarising the Growth Trajectory Using MLMs

Growth trajectories can be modelled using MLMs, where random effects or individual-level residuals represent individuals' underlying growth patterns, and the deviation of observed measures from predicted values is given by individual-specific occasion level residuals [6].

A basic random-slope model for linear change can be written:

$$y_{ij}=\beta_{oj}+u_{oj}+\left(\beta_{1}\times t_{ij}\right)+\left(u_{1j}\times t_{ij}\right)+e_{ij},$$

where *y*_*ij*_ is the height for individual *j* at time *t*_*ij*_, and *e*_*ij*_ ∼ *N* (0, *σ*^2^_*e*__0_) and (*u*_0__*j*_, *u*_1__*j*_) follow a bivariate normal distribution with means of zero, variances *σ*^2^_*u*__0_ and covariance *σ*^2^_*u*__1_.

Here, *β*_0_ and *β*_1_ (the ‘fixed’ coefficients) represent the average intercept and slope, respectively, and *u*_0__*j*_ and *u*_1__*j*_ (the ‘random’ coefficients) represent the deviation from the average intercept and slope, respectively, for individual *j*. The occasion level residuals *e*_*ij*_ represent the measurement error, and here are assumed to have constant variance. This model can be extended to incorporate a complex variance structure at the occasion level [7] by including functions of age and/or other covariates in the function for the occasion level variance. We have not considered this further here, but have found in other applications that considering complex measurement error can improve the fit of the model [8].

The fixed coefficients (*β*_0_ and *β*_1_) and the individual intercept and slope (*u*_0__*j*_ and *u*_1__*j*_) can be used to predict the height for a specific individual at any time point.

### Non-Linear Trajectories

When growth is non-linear, one approach is to find a transformation of either the growth measure or time such that growth is approximately linear [9]. A more flexible method is to include non-linear functions of time in an MLM – although it is then necessary to choose a best-fitting function of time. Restricting the choice of models to a small number of simple polynomials can be misleading, since simple polynomials encompass relatively few curve shapes and do not have asymptotes. More complex polynomials (e.g. cubics and higher powers) may fit badly at extremes of the data and also may produce artefactual turns in the curve shape.

### Fractional Polynomials

An alternative is to select the best-fitting function from a family of flexible polynomial functions [10]. The procedure, known as selection of a fractional polynomial, is described elsewhere [10], so only brief details are given here. With simple (single-level) linear regression, the model deviance of each of eight powers (−2, −1, −0.5, 0, 0.5, 1, 2, 3, where a power of zero is the log function) is used to identify the best-fitting single polynomial. All possible combinations of pairs of these polynomials are then examined, and again the model deviance is used to select the best-fitting model containing two powers. The difference between the model deviance for the best-fitting polynomials of degrees 1 and 2 are then compared to the χ^2^ distribution with 2 degrees of freedom (because one extra power and coefficient are being estimated), to test whether the addition of an extra polynomial term significantly improves the model.

In the multilevel framework, the method is similar, with the model for a polynomial of degree 2 with powers *p*_*1*_ and *p*_*2*_ being given by:

$$\frac{\text{if}p_{1}\neq p_{2}:}{y_{ij}=\beta_{0}+\left(\beta_{1}+u_{1j}\right)t_{ij}^{p1}+\left(\beta_{2}+u_{2j}\right)t_{ij}^{p2}+u_{0j}+e_{ij}.}$$

If *p*_*1*_ = *p*_*2*_ then the second term is multiplied by the log of time, since we cannot have two separate terms containing the same powers of time:

$$\frac{\text{if}p_{1}=p_{2}:}{y_{ij}=\beta_{0}+\left(\beta_{1}+u_{1j}\right)t_{ij}^{p1}+\left(\beta_{2}+u_{2j}\right)t_{ij}^{p2}\log_{ij}+u_{0j}+e_{ij},}$$

where *β*_0_, *β*_1_ and *β*_2_ are the fixed coefficients describing the average shape of the trajectory, and *u*_0__*j*_, *u*_1__*j*_ and *u*_2__*j*_ describe the deviation of individual *j*'s trajectory from this average. The same set of choices of power (−2, −1, −0.5, 0, 0.5, 1, 2, 3, where a power of zero is the log function) are usually used, although some authors also include −3 [11]. The difference in model deviance between the best-fitting polynomial of degree 2 and that of degree 1 is compared to the χ^2^ distribution with 5 degrees of freedom, since one extra power, one fixed coefficient and three random parameters (the variance of one extra random coefficient, *u*_2__*j*_, and its covariance with the other random coefficients, *u*_0__*j*_ and *u*_1__*j*_) are being estimated. As alternatives to the log-likelihood, the Akaike information criterion (AIC) or Bayesian information criterion could be used to select the best-fitting model [11].

When using fractional polynomials, time/age must be strictly greater than zero. Where a trajectory starts at zero (i.e. weight is modelled against age, starting from birth) then a constant can be added to age in order to achieve this strict positivity. However, the fractional polynomials are affected by additive (although not multiplicative) transformations, and thus the choice of this constant is important – one recommendation is to add the smallest difference between successive time measurements [11]. Here we added 0.01 months to all ages. Further analyses could explore the sensitivity to other choices.

We only considered 2nd-degree polynomials here, but 3rd- or higher-degree polynomials may be needed for more complex trajectory shapes (particularly those with several maxima/minima).

### Linear Splines

An alternative approach is to model the growth trajectory as a series of connected lines (‘linear splines’, also called ‘broken-stick’ or ‘piecewise’ models) joined at ‘knots’. For example, a multilevel linear spline model for height with knots at 3 and 6 months would allow different linear slopes from 0 to 3, from 3 to 6, and beyond 6 months, with these slopes varying between individuals.

We define *c* knot points at times *t*_*k*_, *k* = 1, …, *c*, and define *t*_0_ = 0, *t*_*c*__+ 1_ = max(time). For person *j*, with height_*ij*_ observed at time *t*_*ij*_ we create *c* + 1 splines *s*_*ijk*_:

$$\begin{array}{cc} \text{For}k=1,c:s_{ijk}=0 & \text{it}t_{ij}\leq t_{k-1}\\ s_{ijk}=t_{ij}-t_{k-1} & \text{it}t_{ij}\leq t_{k}\\ s_{ijk}=t_{ij}-t_{k-1} & \text{it}t_{ij}>t_{k} \end{array}$$

Thus, each spline is zero for all ages before the knot point at which that spline begins, rises linearly with age until age reaches the next knot point (at which this spline ends), and then takes the value of this 2nd knot point thereafter. In the multilevel context, a model with *c* knots would then be of the form:

$$y_{ij}=\beta_{0}+u_{0j}+\sum_{k=1}^{c+1}\left(\beta_{k}+u_{kj}\right)s_{ijk}+e_{ij},$$

where *β*_*0*_, …, *β*_*c*__+ 1_ are the fixed coefficients describing the average intercept and average slope between each set of knots, *u*_*kj*_ describes the deviation for individual *j* from the average slope between *t*_k – 1_ and t_k_, and *u*_*0j*_ is thedeviation of individual *j*'s intercept from the average intercept.

### Including Individual-Level Covariates

A covariate *X*_*j*_, measured for each person *j*, can be related to growth using either the fractional polynomial or the linear spline models.

For the fractional polynomial model, a term is included for the association of the covariate with the outcome at time zero (in this example, birth length) and its interaction with each of the polynomial terms:

$$\begin{array}{c} \text{if}p_{1}\neq p_{2}:\\ y_{ij}=\beta_{0}+\alpha_{0}X_{j}+\left(\beta_{1}+\alpha_{1}X_{j}+u_{1j}\right)t_{ij}^{p1}+\left(\beta_{2}+\alpha_{2}X_{j}+u_{2j}\right)t_{ij}^{p2}=+u_{0j}+e_{ij};\\ \text{if}p_{1}\neq p_{2}:\\ y_{ij}=\beta_{0}+\alpha_{0}X_{j}+\left(\beta_{1}+\alpha_{1}X_{j}+u_{1j}\right)t_{ij}^{p1}+\left(\beta_{2}+\alpha_{2}X_{j}+u_{2j}\right)t_{ij}^{p2}\log t_{ij}=+u_{0j}+e_{ij}. \end{array}$$

For the linear spline model, a term is included for the association of the covariate with the outcome at time zero, and its interaction with each of the spline terms. These interactions are easily interpretable as being the increase in linear change related to a 1-unit increase in the covariate.

$$y_{ij}=\beta_{0}+\alpha_{0}X_{j}+u_{0j}+\sum_{k=1}^{c+1}\left(\beta_{1}+\alpha_{1}X_{j}+u_{kj}\right)s_{ijk}+e_{ij}.$$

## Illustrative Example: Modelling Height during Infancy and Early Childhood

### Study Population

The Avon Longitudinal Study of Parents and Children (ALSPAC) is a prospective cohort study investigating the health and development of children. Full study methodology has been published elsewhere [12] and is detailed on the study website (www.bristol.ac.uk/alspac). Briefly, pregnant women resident in one of three Bristol-based health districts with an expected delivery date between the 1st of April 1991 and the 31st of December 1992 were invited to take part. Of these, 14,541 women were enrolled, with 14,062 children born, of which 13,988 were alive at 1 year. Mothers who had moved out of the study area, those lost to follow-up and those taking part in another study of infant development were excluded. Follow-up has included parent- and child-completed questionnaires, links to routine data and clinic attendance. A random sub-sample of children from the last 6 months of recruitment [‘Children in Focus’ (CiF) group – approx. 10% of the total cohort] were invited to clinics between ages 4 months and 5 years; all children were invited to clinics from age 7 years onwards. CiF clinics were held at 4, 8, 12, 18, 25, 31, 37, 43, 49 and 61 months, and 1,432 families attended at least one CiF clinic. Ethical approval of the study was obtained from the ALSPAC Law and Ethics Committee and the local research ethics committees. Please note that the study website contains details of all the data that are available through a fully searchable data dictionary (http://www.bris.ac.uk/alspac/researchers/data-access/data-dictionary).

### Measurement of Length and Height

Length/height data for the children are available from several sources. Birth length (crown-heel) was measured for almost the whole cohort by ALSPAC staff who visited newborns soon after birth (median 1 day, range 1-14 days) using a Harpenden neonatometer (Holtain Ltd). From birth to 5 years, measurements are also available for the majority of the cohort from health visitor records, which form part of standard child care in the United Kingdom. At the CiF clinics, crown-heel length was measured up to 25 months using a Harpenden neonatometer and from 25 months standing height was measured using a Leicester height measure (Seca). From age 7-10 years, all children were invited to annual clinic visits, at which standing height (without shoes) was measured to the last complete millimetre using the Harpenden stadiometer (Holtain Ltd). Across all ages, parent-reported child heights are also available from questionnaires. We have previously shown that the health visitor measurements are reliable [13]. A binary indicator of parent-reported measurements versus research or clinical record measurements was included in all growth trajectory models as a fixed effect.

### Assessment of Social Class

Mother and her partner's occupations (self reported in questionnaires at baseline) were used to generate a measure of the highest household social class, using the 1991 classification of the UK Office of Population Censuses and Surveys (classes I-V, with III split into manual and non-manual). This was dichotomised into manual and non-manual.

### Analysis Dataset

Because we hypothesised that boys and girls would have different growth trajectories, we have only included girls in the analyses presented here to simplify our description of the methods. We have included in our analyses the 5,588 girls who have 1 or more measures of height between birth and age 10 years and also have data on household social class.

## Results

There were 6,733 girls with at least 1 measure of height; 5,588 of these also had data on social class; 1,097 (19.6%) of the girls were in the manual social class category. The median number of measures per girl was 7, interquartile range 5-10. Table 1 shows the average height for different age groups, both for the entire sample of 5,588 girls and separately by manual and non-manual social class.

### Fractional Polynomials

A MLM was fitted to all available measures of height up to age 10 for all 5,588 girls in the sample, with 0.01 months added to all ages (to ensure that age was strictly positive). The best-fitting fractional polynomial had powers of the square root of age, and the square root of age multiplied by the log of age. We assumed an unstructured variance/covariance matrix (shown in online suppl. table 1; see www.karger.com/doi/10.1159/000362695 for all online suppl. material), thus estimating all six variances/covariances for the three random effects. The AIC for this model was 213,683.9. The fixed part of the equation for this best-fitting polynomial was:

$${\text{height}}_{ij}=\beta_{0}+\beta_{1}{\left(t_{ij}\right)}^{0.5}+\beta_{2}\left[\log\left(t_{ij}\right)\times {\left(t_{ij}\right)}^{0.5}\right],$$

where:

$$\begin{array}{c} t_{ij}=\text{age}\left(\text{months}\right)+0.01\text{at time}i\text{forindividual}j\\ \beta_{0}=49.69\left(\text{SE}=0.044\right)\\ \beta_{1}=5.93\left(\text{SE}=0.023\right)\\ \beta_{2}=0.452\left(\text{SE}=0.005\right). \end{array}$$

**Fig. 1:**
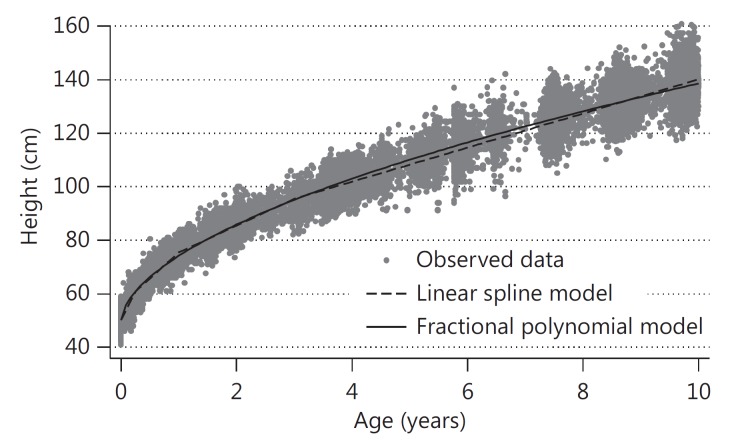
Average predicted height trajectories from birth to 10 years in girls from the ALSPAC study predicted by the best-fitting fractional polynomial and the linear spline MLMs.

The interpretation of these coefficients is difficult due to the complexity of the function – a graph is the clearest way to examine the average curve for this best-fitting polynomial (fig. 1). There was no evidence of any peaks or sharp changes in the velocity of height gain. The fixed coefficients, plus the individual-level coefficients for the intercept, square root of age and the square root of age multiplied by the log of age terms were used to predict height for each individual, for each age at which they were measured. The means of these predicted heights (from the best-fitting fractional polynomial) in each age period are shown in table 2 together with the average observed height during that period. For example, the average predicted height for all children aged 0-3 months was 57.43 cm, and 95% of the predicted heights were within −3.6 to +2.0 cm of the observed height. The model appears to fit the data well, with 95% limits of agreement between observed and expected heights being within 10% of the mean height in each period.

### Linear Spline Model

From examination of the best-fitting fractional polynomial and previous work [14], it appeared that height gain was fastest in the first few months of life, slowing down thereafter, with approximately linear growth between 0-3, 3-12, 12-36 and 36-120 months (with a different linear rate in each of these time periods). Models with knot points at each whole month within 6 months of either side of these knot points (or within 3 months for the 1st knot point) were compared: we fitted models with 2 knot points only and compared the best fitting of these with the best-fitting model with 3 knot points. The best-fitting model based only on the model deviance had knots at 2, 11 and 32 months. For ease of interpretation and clarity of presentation, we compared this model with one where knot points were placed at 3, 12 and 36 months. There was very little change in model deviance and no change in the mean and range of differences between observed measurements and those predicted by the MLM, so the model with knot points at 3, 12 and 36 months was chosen. The AIC for this model was 210,887.1 – lower than that of the fractional polynomial model, indicating slightly better model fit. We assumed an unstructured variance/covariance matrix (shown in online suppl. table 2), thus estimating all 15 variances/covariances for the 5 random effects. The fixed part of this model had the form:

$$height_{ij}=\beta_{0}+\beta_{1}s_{ij1}+\beta_{2}s_{ij2}+\beta_{3}s_{ij3}+\beta_{4}s_{ij4},$$

where:

$$\begin{array}{c} \beta_{0}=50.09\left(\text{SE}=0.040\right)\text{cm}\\ \beta_{1}=3.57\left(\text{SE}=0.014\right)\text{cm/month}\\ \beta_{2}=1.63\left(\text{SE}=0.006\right)\text{cm/month}\\ \beta_{3}=0.83\left(\text{SE}=0.002\right)\text{cm/month}\\ \beta_{4}=3.53\left(\text{SE}=0.001\right)\text{cm/month}. \end{array}$$

The interpretation of these coefficients is simple (in contrast to those from the fractional polynomials). Estimated average length at birth is 50.09 cm, with estimated growth of 3.57 cm/month during the period from birth to 3 months, and rate of change in height decreasing with age to a mean gain of 0.53 cm/month after age 3 years.

The average shape for this trajectory (given by the fixed coefficients) is shown in figure 1. The fixed coefficients, plus the individual-level coefficients for each slope, were used to predict height for each individual, for each age at which they were measured. The average of the predicted heights in each age period are shown in table 2. The model appears to fit the data well, with 95% limits of agreement between observed and expected heights being within 10% of the mean height in each period. Table 2 also shows that, on average, the linear spline model fits the observed data slightly better than the fractional polynomial model, as the mean and the range of the differences between observed and predicted heights are smaller for the linear spline model.

### Including Social Class as an Individual-Level Covariate

**Fig. 2:**
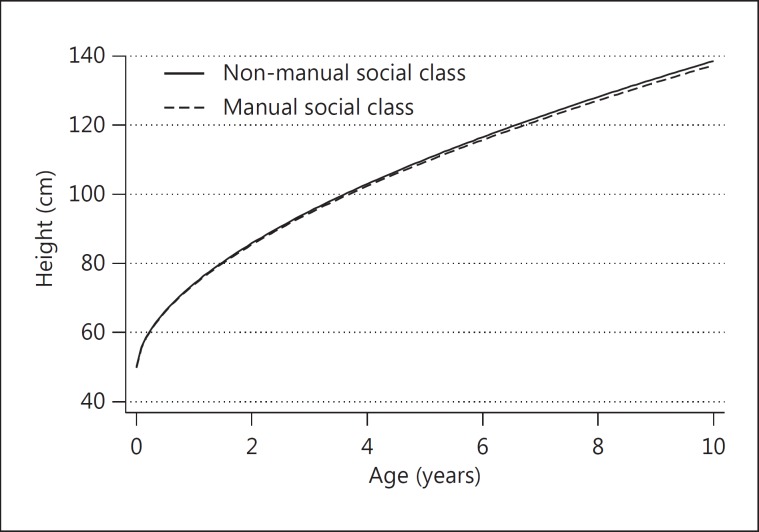
Average predicted height gain from birth to 10 years in girls of the manual and non-manual social class from the ALSPAC study predicted by the best-fitting fractional polynomial model.

In order to assess the association between household social class and child growth, social class (as a binary covariate: manual vs. non-manual) was included in both the fractional polynomial and the linear spline models. For the fractional polynomials, the coefficients for the interaction between social class and the powers of age are not readily interpretable. A plot of the predicted average height gain in girls of the manual and non-manual social class (fig. 2) shows that there is a gradually widening difference: girls of the non-manual social class are slightly taller by 10 years of age. A table of predicted heights at different ages (table 3) reveals that the initial difference in height between the two groups decreases from birth to approximately 1 year of age, and then increases.

**Fig. 3:**
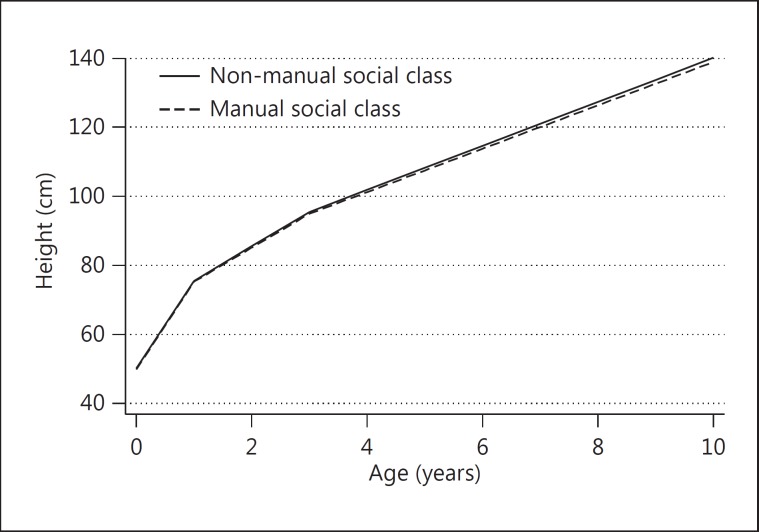
Average predicted height gain from birth to 10 years in girls of the manual and non-manual social class from the ALSPAC study predicted by the best-fitting linear spline model.

For the linear spline model, the coefficients for the interactions between social class and the spline terms show the predicted growth rates for each social class group (table 4). These indicate that girls of the manual social class have lower birth length and slower growth than those of the non-manual social class, except between birth and 3 months, when the manual group grows 0.087 (95% CI 0.016-0.158) cm/month faster. The plot of the predicted average height gain (fig. 3) and tables of predicted heights at different ages (table 3) are very similar to those from the fractional polynomial model.

## Discussion

We have used MLMs to estimate height gain trajectories for each individual, comparing the fractional polynomial and linear splines approaches. The fractional polynomial model assumes a smooth, monotonic curve of height with age, whereas the linear spline model assumes a biologically implausible (but more interpretable) piecewise linear relationship between height gain and age. Both models demonstrated an initially rapid growth in height, with rate of growth decreasing over time. Both models also showed that girls in the manual social class gained height more slowly than those in the non-manual social class. Despite the implausible linearity assumption, the 3-knot linear spline model appeared to fit the height data slightly better than the two-degree fractional polynomial in our example.

Fractional polynomials have been compared to conventional polynomials in the multilevel setting [11]. Using simulated data, fractional polynomials were more parsimonious than conventional polynomials, with at least equal fit to the data. Fractional polynomials have been used in the multilevel modelling framework to model body mass index [15], early growth [16], sodium content in breast milk [17] and blood pressure in pregnancy [18]. The effect of covariates on trajectories can be explored, and summaries of the curves can be extracted – for example, curves can be differentiated to obtain age at maxima/minima [11,15]. An a priori decision is often made only to examine fractional polynomials up to degree 2. Degree 1 fractional polynomials are strictly monotonic, whereas those of degree 2 can be non-monotonic, with 1 maximum or minimum. The choice of degree to consider should be driven by theory of underlying biology. For example, for height change over childhood, degree 2 is a sensible choice, whereas for body mass index in infancy/puberty, with multiple potential maxima/minima, degrees greater than 2 should be included [15].

Linear spline models have been used to model growth in childhood [14,19,20], blood pressure in pregnancy [21,22], aspects of gait in childhood [23] and fetal growth [16]. Linear spline models are often used where the knot point is known – for example, to model body fat before and after menarche [24]. Where the number and placing of the knot point(s) are unknown, they must be estimated. For simple regression models, options for selecting the number and position of the knots include: using a large number of knots and reducing the number until a ‘smooth’ curve is reached; placing knots at centiles of the time variable, and stepwise regression to select those knots which are ‘significant’ [25]. We are aware of no such guidance for MLMs; the theoretical properties of these methods are unknown. We have previously used fractional polynomials to derive a smooth approximation to the curve, and used its derivatives to inform the number and placing of knot points [14,19]. External knowledge of growth patterns or ‘critical’ periods throughout the life course may also inform the placement of knots.

A comparison of fractional polynomials and linear splines in a non-repeated measure setting showed that the two approaches performed similarly, although there was a tendency for fractional polynomials to perform better at recovering simpler functions and splines for more complex functions [26]. Multilevel linear splines performed better than fractional polynomials in modelling repeated measures of prostate-specific antigen over time, although smoothing methods outperformed both [27]. The number of parameters in a multilevel linear spline model can become large [28], particularly if each spline has an associated random effect. With balanced data, to fit a fractional polynomial with random effects for all parameters requires at least 3 measures per individual for degree 1, and 4 for degree 2. To fit a linear spline model with 1 knot point would require at least 4 measures per individual, with 5 required for a 2-knot-point model. Table 5 outlines some of the differences between the two methods.

Alternative models include fitting individual knots in a linear spline model [29], using non-linear splines [30], more complex transformations [31] or non-linear functions (including the Preece-Baines model [32,33]). However, these suffer from needing a larger number of observations per individual (e.g. Preece-Baines estimates 5 parameters per person), are harder to fit in general multilevel modelling software and suffer from the same difficulty in interpretation as the fractional polynomial models described above.

## Conclusions

Either fractional polynomials or linear splines can be used to summarise growth and to identify relationships between individual-level exposures and growth. Linear splines may be more easily interpretable, particularly when examining the associations between exposures and growth. The choice of method depends upon the aim of the study, the number of measures available per individual and the complexity of the underlying trajectories.

## Disclosure Statement

K.T. was invited to attend an International Research Workshop on Analysis of Child Growth Trajectories at which this work was presented. Travel expenses and an honorarium were paid. The workshop was organized by Professor Berthold Koletzko, Dr. von Hauner Children's Hospital, Ludwig Maximilians University of Munich Medical Centre, Munich, Germany (LMU) and the Early Nutrition Academy. The workshop was supported by the Centre for Advanced Studies LMU, the German Research Council (DFG), and unrestricted educational grants from Abbott Nutrition and the International Life Sciences Institute.

LDH is a co-applicant on a grant from the Nestlé Foundation that includes analysis of child growth.

None of the remaining authors has a conflict of interest to declare.

## Supplementary Material

Supplementary table

## Figures and Tables

**Table 1:** Ages and heights of 5,588 girls up to 10 years of age and with complete social class data: ALSPAC Study

Growth period	Measures, n	Mean height, cm (SD)			Mean difference in height, cm (95% CI) manual – non-manual social class
		entire group	non-manual social class	manual social class	
Birth	4,326	50.29 (2.24)	50.37 (2.23)	49.97 (2.24)	−0.40 (−0.57 to −0.23)
0–3 months	5,789	56.64 (2.95)	56.69 (2.97)	56.40 (2.86)	−0.30 (−0.50 to −0.10)
3–12 months	8,100	68.97 (4.66)	68.92 (4.71)	69.27 (4.40)	0.35 (0.07 to 0.64)
1–3 years	7,708	82.95 (5.67)	83.03 (5.68)	82.53 (5.60)	−0.51 (−0.89 to −0.13)
3–10 years	20,187	117.84 (15.27)	118.12 (15.26)	116.3 (15.27)	−1.86 (–2.51 to −1.21)

**Table 2:** Fit of the best-fitting fractional polynomial and linear spline models to data on height from birth to10 years of age in 5,588 girls: ALSPAC Study 1990–2002

Growth period	Mean observed height, cm (SD)	Fractional polynomial model (AIC = 213,683.9)			Linear spline model (AIC = 210,887.1)		
		mean predicted height	mean difference (observed – predicted)	95% limits of agreement^a^	mean predicted height	mean difference (observed – predicted)	95% limits of agreement^a^
Birth	50.29 (2.24)	50.01	0.28	−1.63 to 2.20	50.38	−0.09	−1.79 to 1.61
0–3 months	56.64 (2.95)	57.43	−0.79	–3.62 to 2.04	56.50	0.14	–2.30 to 2.57
3–12 months	68.97 (4.66)	68.58	0.39	–2.41 to 3.19	69.02	−0.05	–2.37 to 2.26
1–3 years	82.95 (5.67)	82.38	0.57	–2.66 to 3.79	82.84	0.11	–2.97 to 3.18
3–10 years	117.84 (15.27)	118.06	−0.22	–3.76 to 3.32	117.87	−0.03	–3.33 to 3.28

a Limits within which 95% of the differences between observed and predicted values lie.

**Table 3:** Predicted height per linear spline period in girls from the non-manual social class, manual social class, and the mean difference in predicted height comparing girls from the manual with the non-manual social class

	Mean height, cm (SD)		Mean difference in height, cm (95% CI) manual – non-manual social class
	non-manual social class	manual social class	
Fractional polynomial model			
Birth	49.786 (1.532)	49.299 (1.516)	−0.487 (−0.669 to −0.306)
3 months	60.897 (1.655)	60.651 (1.682)	−0.246 (−0.386 to −0.106)
1 year	74.198 (2.081)	73.920 (2.098)	−0.278 (−0.445 to −0.110)
3 years	95.103 (3.053)	94.604 (3.013)	−0.499 (−0.728 to −0.270)
10 years	138.614 (5.754)	137.355 (5.530)	−1.259 (−1.723 to −0.795)
Linear spline model			
Birth	50.173 (1.642)	49.734 (1.652)	−0.439 (−0.599 to −0.279)
3 months	60.833 (1.921)	60.654 (1.958)	−0.179 (−0.385 to 0.028)
1 year	75.466 (2.202)	75.267 (2.233)	−0.199 (−0.410 to 0.011)
3 years	95.529 (3.287)	94.958 (3.238)	−0.570 (−0.850 to −0.291)
10 years	140.167 (5.745)	138.948 (5.476)	−1.219 (−1.709 to −0.729)

**Table 4:** Mean growth rates per linear spline period in the non-manual social class and the mean difference in growth rates comparing the manual with the non-manual social class

	Mean growth rate (SD) non-manual social class	Mean difference (95% CI) manual vs. non-manual social class
Birth length, cm Growth rate, cm/month	50.173 (1.642)	−0.439 (−0.599 to −0.279)
0–3 months	3.553 (0.241)	0.087 (0.016 to 0.158)
3–12 months	1.626 (0.136)	−0.002 (−0.030 to 0.026)
1–3 years	0.836 (0.071)	−0.015 (−0.026 to −0.005)
3–10 years	0.531 (0.035)	−0.008 (−0.013 to −0.003)

**Table 5:** Comparison of fractional polynomials and splines

Fractional polynomial	Linear splines
Automated procedure for selecting the best-fitting model	‘Rule of thumb’ – no consensus on the best method to choose the number and position of knot points

Needs constant term (fixed and random effect), plus 1 fixed and 1 random effect per degree (e.g. a 2nd-degree polynomial needs 3 fixed effects and 3 random effects)	Needs constant term (fixed and random effect), plus 1 fixed and 1 random effect, plus 1 fixed and random effect per knot point (e.g. a model with 2 knot points requires 4 fixed and 4 random effects)

Smooth curve	Biologically implausible broken-stick model

Harder to interpret coefficients directly	Easy to interpret coefficients
