# Supplementary material for: Modelling Childhood Growth Using Fractional Polynomials and Linear Splines
Source: Ann Nutr Metab. 2014 Nov 18;65(3):129–38. doi: 10.1159/000362695 (PMC4264511; doi:10.1159/000362695)
Supplement: Supplementary file 1 — Supplementary table [file anm-0065-0129-s01.doc]

**Supplementary Material**

Supplemental Table 1 – Variances (diagonal elements) and covariances (off-diagonal elements) , with standard errors, between the individual-level random effects in the Fractional Polynomial Model, and their standard errors.

|  | Intercept | Age ^0.5^ | Log(Age)*Age ^0.5^ |
| --- | --- | --- | --- |
| Intercept | 3.8307  (0.1426) |  |  |
| Age ^0.5^ | -0.7881  (0.0729) | 0.8830  (0.0519) |  |
| Log(Age)*Age ^0.5^ | 0.1720  (0.0151) | -0.1698  (0.0107) | 0.0447  (0.0023) |

Supplemental Table 2 – Variances (diagonal elements) and covariances (off-diagonal elements) between the individual-level random effects in the Linear Spline Model, and their standard errors.

|  | Intercept | 0-3 m | 3-12 m | 12-36 m | 36+ m |
| --- | --- | --- | --- | --- | --- |
| Intercept | 3.6977  (0.1135) |  |  |  |  |
| 0-3 m | -0.0907  (0.0376) | 0.1919  (0.0203) |  |  |  |
| 3-12 m | -0.1102  (0.0129) | 0.0015 (0.0057) | 0.040  (0.0028) |  |  |
| 12-36 m | 0.0188  (0.0050) | -0.0023  (0.0021) | 0.0018  (0.0008) | 0.0090  (0.0004) |  |
| 36+ m | 0.0168  (0.0022) | 0.0019  (0.0009) | 0.0015  (0.0004) | 0.0013  (0.0001) | 0.002  (0.0001) |
